# Comparative Analysis of Energy Use and Greenhouse Gas Emission of Diesel and Electric Trucks for Food Distribution in Gowanus District of New York City

**DOI:** 10.3389/fdata.2021.693820

**Published:** 2021-07-26

**Authors:** Raghul Elangovan, Ondrea Kanwhen, Ziqian Dong, Ahmed Mohamed, Roberto Rojas-Cessa

**Affiliations:** ^1^ Networking and Innovation Laboratory, Department of Electrical and Computer Engineering, College of Engineering and Computing Sciences, New York Institute of Technology, New York, NY, United States; ^2^ Smart Grid Interdependencies Laboratory, Department of Electrical Engineering, City University of New York City College, New York, NY, United States; ^3^ Networking Research Laboratory, Department of Electrical and Computer Engineering, New Jersey Institute of Technology, Newark, NJ, United States

**Keywords:** food distribution, electric trucks, energy, greenhouse gases, electrification, freight, sustainability

## Abstract

New York City’s food distribution system is among the largest in the United States. Food is transported by trucks from twelve major distribution centers to the city’s point-of-sale locations. Trucks consume large amounts of energy and contribute to large amounts of greenhouse gas emissions. Therefore, there is interest to increase the efficiency of New York City’s food distribution system. The Gowanus district in New York City is undergoing rezoning from an industrial zone to a mix residential and industrial zone. It serves as a living lab to test new initiatives, policies, and new infrastructure for electric vehicles. We analyze the impact of electrification of food-distribution trucks on greenhouse gas emissions and electricity demand in this paper. However, such analysis faces the challenges of accessing available and granular data, modeling of demands and deliveries that incorporate logistics and inventory management of different types of food retail stores, delivery route selection, and delivery schedule to optimize food distribution. We propose a framework to estimate truck routes for food delivery at a district level. We model the schedule of food delivery from a distribution center to retail stores as a vehicle routing problem using an optimization solver. Our case study shows that diesel trucks consume 300% more energy than electric trucks and generate 40% more greenhouse gases than diesel trucks for food distribution in the Gowanus district.

## 1 Introduction

New York City’s food distribution system is among the largest in the United States. Food is transported from twelve major distribution centers to various consumer point-of-sale locations. Trucks are the major means of food transportation in New York City (NYC) NYC (2016). They are also large consumers of energy and generators of greenhouse gases (Salvatore et al., 2017). In the United States, the transportation sector contributes to 28% of the total energy consumed each year NASEM (2021), 58% of which is from light trucks, cars, and motorcycles, 23% is from other trucks, and the remaining 19% is from aircrafts, boats, ships, trains, buses, and pipelines. With New York City’s strategic plan to reach carbon neutral by 2050 NYC (2019), the city has experimented electrification of the Department of Sanitation truck fleet and Metropolitan Transportation Authority (MTA) buses Chang (2020), Guse (2020). There is interest in understanding the impact of electrification of the food distribution system in the city.

Food industries comprise mostly private sector businesses in the U.S. Therefore, data on the last-mile food deliveries from distribution centers to local stores are often unpublished. This lack of data is a key challenge for case studies that analyze the carbon footprint of food distribution systems. The question is how can we leverage existing open data to estimate last-mile food deliveries from distribution centers to retail stores to understand the impact on energy use and greenhouse gas (GHG) emissions when switching to electric fleet.

Here, we present a case study on the impact of electrification of food transportation fleet on energy consumption and GHG emissions on a district level. As this level of urban renewal and planning has an easier adoption for innovative solutions for sustainable design, our goal is to use this case study to provide a procedure for various stakeholders and decision makers to evaluate the potential impact of policy implementation on their carbon footprint goals. Our case study targets the Gowanus district in NYC. This district is undergoing rezoning from an industrial zone to a mix residential and industrial one. Therefore, it provides a great opportunity for testing new initiatives such as planning for infrastructure supporting electric vehicles.

As a more realistic scenario in this comparative study, we develop a framework to estimate the district-level food demand, the number and type of food-delivery trucks, and the scheduled route for each truck. We use open access data on food distribution centers of NYC and retail stores in the Gowanus district. To address the data availability gap, we adopt a methodology on urban food distribution at the Pacific southwest to estimate the food demands at retail stores McCormack et al. (2010). The store demand and delivery frequencies are determined based on the store type and size and the type of truck. We consider the life cycle (including the manufacture and operation phases) of the electric and diesel vehicles in analyzing their energy demand and GHG emissions. The delivery route for each truck from a distribution center to the selected stores are estimated using Google Operation Research (OR) to minimize the total distance traveled by each truck Google (2020). We compare energy consumption and GHG emissions for electric and diesel trucks to deliver food from a distribution center to the stores at the Gowanus district. Our case study results show that diesel trucks consume 300% more energy and emit 40% more greenhouse gases than electric trucks to provide food distribution service for this district.

The contributions of this paper are:

• We present a comparative analysis of a district-level food distribution in NYC using electric and diesel trucks.
• We propose a framework to evaluate the impact of using electric trucks in last-mile food distribution which includes the segment from distribution centers to retail stores.
• We show the impact on energy consumption and GHG emission of diesel and electric trucks to make last-mile food deliveries in the Gowanus district of NYC.

## 2 Related Works

Last-mile food distribution is a complex logistics scheduling problem. This analysis requires to consider food demand, number of trucks, types of trucks and route selection (distance, duration and number of stops). Such data are often private and not published. Elangovan and Dong (2020) used the data for the different types of food requirements in the U. S. obtained from a United States Department of Agriculture (USDA) survey to estimate daily food demand in kilogram (kg) for the Gowanus district in NYC. (Goodchild and Ukrainczyk, 2016) showed that interviews, surveys and onsite observations provide a reliable and accurate method for acquiring the necessary food delivery distribution truck data.

Liimatainen et al. (2019) analyzed the electric vehicle potential across commodities in Switzerland and Finland and found that while medium duty short-haul trucks are already competitive when compared to the diesel alternative, there are only some circumstances where heavy duty long-haul trucks are both economically viable and ecologically sustainable. The difference in competitiveness is primarily due to whether the limitation of cargo capacity is based on volume or weight. As electric trucks carry up to 80% the weight that diesel trucks do, more electric trucks may be needed to meet the same service demands. Gao et al. (2018) estimated that Classes 7 and 8 heavy-duty vehicles using a 250–600 kWh battery capacity results in as much as 3,500 kg mass penalty (trucks overweight penalty). However, if the load of the truck is reduced, the size of the battery needed is reduced and therefore it reduces the mass penalty. However, Classes 3 through 6 vehicles do not have a similar mass penalty as they require less powerful and smaller batteries, so that they are considered a more viable option for electrification.

A comparative study of medium duty delivery electric trucks (Classes 4–6; 6–12 ton gross vehicle weight) against diesel trucks was conducted by Feng and Figliozzi (2013). It showed that electric trucks are economically competitive in high utilization scenarios, especially when the battery needs no replacement over the lifetime of the electric truck. Lee et al. (2013) estimated that the total cost of ownership (TCO) of electric delivery trucks is 22% less than that of diesel trucks in their life cycle. While refrigeration may be needed for food transportation such as meat, dairy, fruits, etc., the additional engine for transportation refrigeration units (TRUs) may also need to be considered for electrification. Thornton et al. (2018) showed that switching from TRUs to Electric Transport Refrigeration Unit (eTRUs) produces measurable cost savings for businesses, and the total amount of savings depends on the business’ current operational practices.

Lee et al. (2013) compared electric and diesel delivery trucks in terms of life-cycle energy consumption, greenhouse gas emissions, and total cost of ownership. The relative benefits of electric trucks depend heavily on the vehicle efficiency associated with drive cycle, diesel fuel price, travel demand, electric drive battery replacement and price, electricity generation and transmission efficiency, electric truck recharging infrastructure, and purchase price. A similar study by Bates et al. (2018) analyzed the importance and impact of the last-mile delivery by freight. Using the data from the field work and quantitative analysis of freight and location data, the authors identified 10 aspects that can be optimized with technology, such as route selection, loading sequence, and others.

Kurien and Srivastava (2020) showed that electric vehicles have lower carbon emissions than combustion-engine vehicles even after considering the indirect carbon emissions from electricity generation. Yang et al. (2020) presented a comprehensive life cycle assessment of the fuel cell vehicle, electric vehicle and internal combustion engine vehicle in China and compared their sustainability under different hydrogen production methods and driving mileages.

Zhou et al. (2017) studied the life cycle GHG emissions and lifetime costs of medium-duty diesel and battery electric trucks in Toronto. They found that the lifetime cost of the Battery Electric Trucks can be lower than that of the diesel truck under driving conditions with frequent stops/starts and with low payloads and low battery and charging station costs. These variables also affect the estimated GHG abatement costs, which are highly relevant as carbon pricing is being introduced in the province. Zhao et al. (2016) analyzed the environmental impacts of various alternative delivery trucks including battery electric, diesel, diesel-electric hybrid, and compressed natural gas trucks from the regular trucks. Shi et al. (2019) presented a cradle-to-grave assessment of energy consumption, CO2 emissions and emissions of electric vehicles in Hebei Province, China. The analysis addressed both the fuel life cycle and vehicle life cycle for conventional gasoline and battery electric vehicles. The study by Liimatainen et al. (2019) also showed that electric trucks are already a feasible solution for a large share of road freight haulage with medium duty trucks. Improvements to battery capacity and recharging infrastructure may also make electric trucks a viable option to substitute heavy duty rigid trucks and semitrailers. It also can create a huge impact on the electric grid.

Others conducted life cycle assessment to evaluate the environmental impacts in various stages of the food supply chain Elsoragaby et al. (2019), Mostashari-Rad et al. (2019), Saber et al. (2020), Khanali et al. (2021), Mostashari-Rad et al. (2021), Lv et al. (2019) Nabavi-Pelesaraei et al. (2019). Ghasemi-Mobtaker et al. (2020). These studies further demonstrate that energy use and greenhouse gas emissions analysis of a food supply chain is highly dependent on the location as well as the adopted practice and technology.

## 3 Case Study of Food Distribution for Gowanus District in NYC

Hunts Point food distribution center is one of the largest in the United Sates. It serves the New York Metropolitan area NYCEDC (2016). It is located in Bronx, NYC, with approximately over 155 public and private wholesalers and suppliers, and accounts for an estimated 60% of the produce sales in New York City.

### 3.1 Data Curation

The data set used in this case study includes information on retail food stores. It is provided by the New York State Department of Agriculture and Markets on the Open Data NY website (Division of Food Safety and Inspection, 2020). The data set covers the retail stores in New York state, and includes information of establishment type, the business licenses and fees, store size in square feet, and location. An alternative data source that can be considered is the Commodity Flow Survey, which is conducted every five years by the U. S. Census Bureau and the Bureau of Transportation.

### 3.2 Methodology for Estimation of Last-Mile Delivery by Trucks

To estimate the last-mile food deliveries from food distribution center to retail stores, we break down the framework into three stages: retail store demand estimation, truck delivery estimation, and truck route estimation.

#### 3.2.1 Retail Store Demand Estimation

We first map the distributors with the stores based on the products they carry because each product may be supplied by different distributors.

##### 3.2.1.1 Distributor and Store Mapping

**FIGURE 1:**
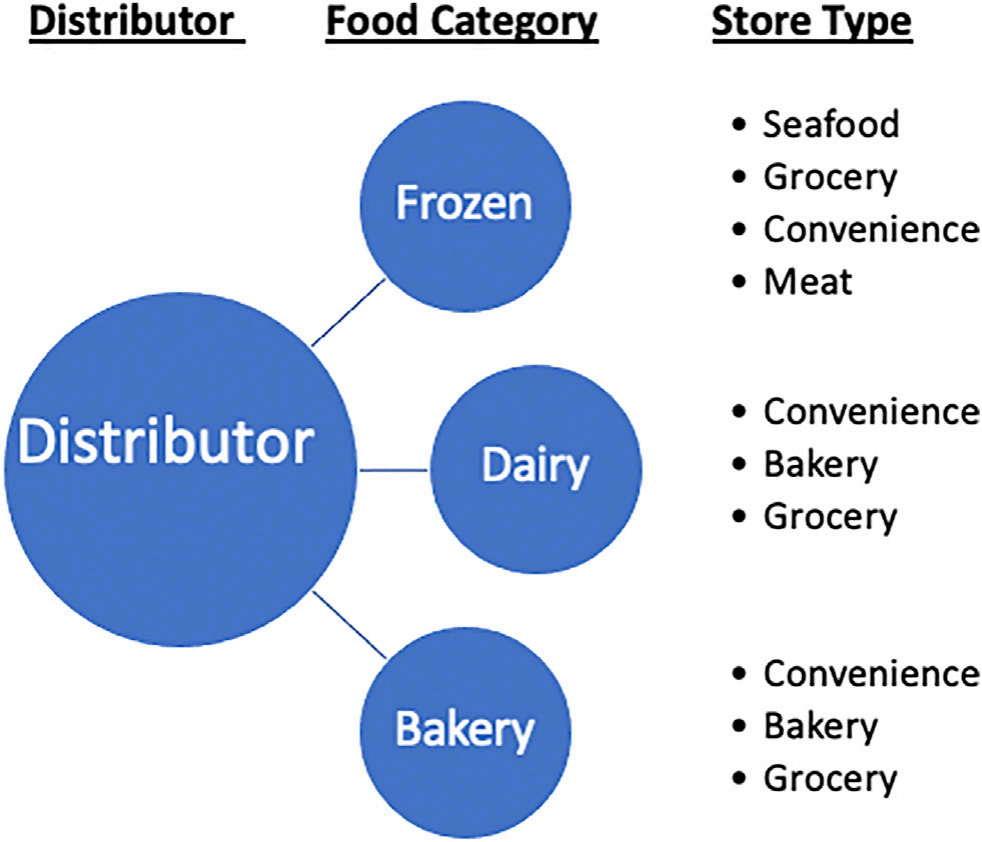
Diagram of the relationship between a distributor and the store types via the corresponding food categories.

We created a relationship map based on the categories of food sold at different stores and the product categories delivered by distributors, as shown in Figure 1.

We used the following terms in the estimation of store demand:

• Store: A retail food store located in the Gowanus district.
• Store Type: The type of food a store sells, as described by the data set provided by the New York Department of Agriculture and Markets.
• Food Category: The type of food items a distributor may supply including frozen food, dairy product, and bakery.
• Distributor: A distributor is a company or a wholesaler that provides food and non-food products to a group of point-of-sales entities such as restaurants, retail stores, schools, etc. A distributor may carry one or multiple food categories.

The adopted product categories reflect the type of stores in the Gowanus district. This categorization can be customized for other regions based on their regional food demands. We made the following assumption in the distributor and store mapping:

• The distributors in the Hunts Point Food Distribution Center are representative of other distributors that also supply the stores in the Gowanus district.
• A store receives a mixture of different food products.
• Stores use third party food delivery services.

##### 3.2.1.2 Methodology

**FIGURE 2:**
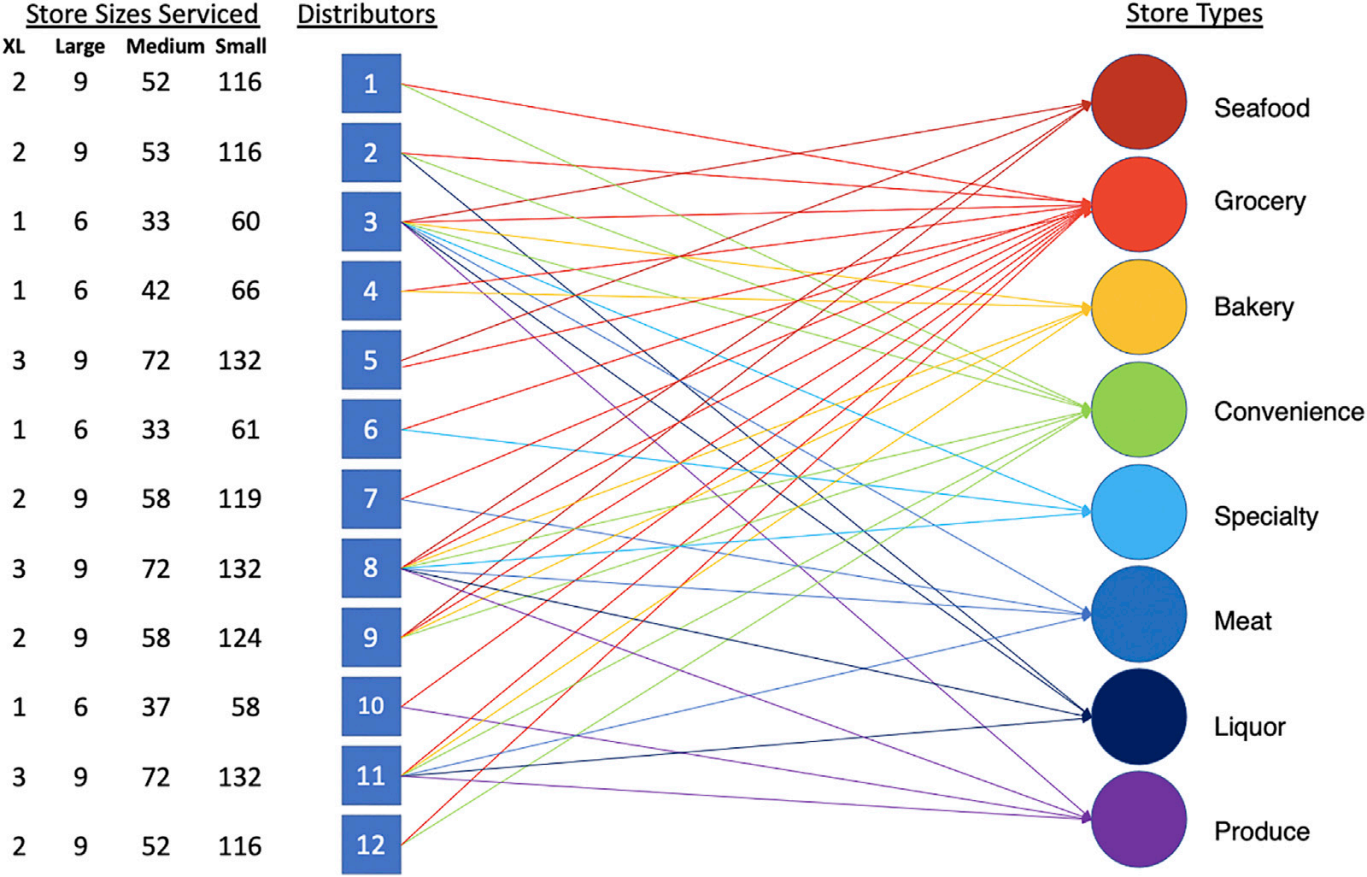
Distributors mapped to each store type along with the number and size of the stores serviced.

Stores are categorized into: Bakery, Convenience, Grocery, Meat, Seafood, and Specialty. Each store type is mapped to one or multiple food categories. A single distributor carries one or multiple food categories. The distributors and stores have a many-to-many relationship. A Standard Query Language (SQL) query was used to map the distributors to stores to provide a list of stores that each distributor services and the number of food delivery trucks from each distributor. Figure 2 shows how distributors are mapped to each store type along with the number and size of each store serviced.

#### 3.2.2 Truck Delivery Estimation

To estimate the number of trucks and types of trucks needed to service the Gowanus district, we used the observatory truck delivery data in the Pacific Southwest (Seattle and Puget Sound region) by McCormack et al. (2010) to extrapolate the amount of truck deliveries for our case study. This extrapolation includes estimating the number of trucks needed to deliver food products to a store and the time a truck spent at a store. Due to the regional demographic and land use differences between NYC and the Seattle regions, the distribution and types of food retail stores in these two regions are also different. It is observed that the stores in the Seattle region have more square footage (23,000 ft) than those in the Gowanus district. Gowanus has stores as small as 200 ft, with 98% of stores under 23,000 ft (210 or 214 stores), and 62% of stores under 2000 ft. Other methodologies that can be used for estimating the number of truck deliveries include using the actual food consumption data or survey data from both food distributors and retail stores on the details of their truck deliveries. The pros and cons of these methodologies are listed in Table 1. To adapt the observational data to our case study, we made the following assumptions:

**TABLE 1:** Methodologies considered for estimating the number of food delivery vehicles.

Methodology	Pros	Cons
1. Use the amount (size and weight) of food consumed to determine the number of trucks needed	• Food consumption and vehicle weight data readily available	• How to accurately allocate food to each truck taking into consideration volume and weight capacity
		• How to account for food waste at retail locations
2. Conduct surveys of food distributors as well as retail locations to understand the number and the types of trucks being used	• Most accurate method for determining truck category, quantity and frequency	• Resource intensive
		• Time consuming
3. Estimate the Gowanus distribution truck numbers using survey and observational data	• Accurate and relevant data observed first hand	• The data is for a different market than the one being studied in this paper
		• Assumptions needed to estimate demands for different size store

• The number of deliveries is proportional to the size of the store.
• Population size does not affect the amount of products stocked by a store.

The methodology to estimate the number of delivery vehicles needed to service Gowanus on a daily basis is outlined in Algorithm 1. The attributes and their definitions are listed in Table 2.

**Algorithm 1:** Determining the number of delivery trucks.

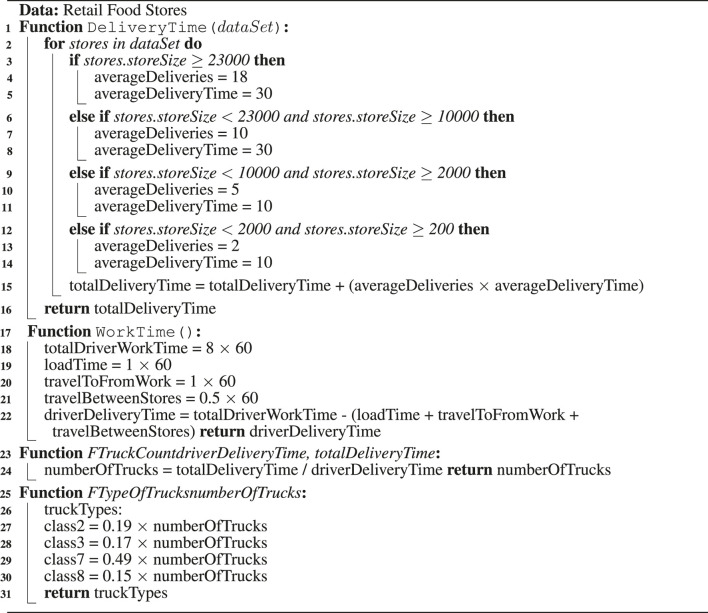

**TABLE 2:** List of definitions and attributes used in Algorithm 1.

Name	Definition	Attributes
driverDeliveryTime	The amount of time a truck spends delivering food in the Gowanus district during a work day	Varies based on the number of stores serviced
driverWorkTime	The total amount of time per day that a driver spends driving. This is estimated to be 8 h	Components of driverWorkTime
		• loadTime: The amount of time a driver spends loading goods at the warehouse. This is estimated to be 1 h
		• travelToFromWork: Average travel time between Gowanus and Hunts Point. This is estimated to be 1 h round trip
		• travelBetweenStores: Total aggregated travel time between stores. This is estimated to be half an hour
stores	Food retail location in Gowanus NYC (2016) that are 200 ft^2^ and larger. Each store requires deliveries	214
storeSize	The square footage of a store. This data is used to categorize stores	• small: 200 ft^2^ ≤ storeSize < 2000 ft^2^
		• medium: 2000 ft^2^ ≤ storeSize < 10,000 ft^2^
		• large: 10,000 ft^2^ ≤ storeSize < 23,000 ft^2^
		• extra-large: storeSize ≥ 23,000 ft^2^
averageDeliveries	The number of trucks that deliver food to a store	• small: averageDeliveries = 8
		• medium: averageDeliveries = 9
		• large: averageDeliveries = 16
		• extra-large: averageDeliveries = 18
averageDeliveryTime	The amount of time a truck spends at each store	• small: averageDeliveryTime = 10
		• medium: averageDeliveryTime = 10
		• large: averageDeliveryTime = 30
		• extra-large: averageDeliveryTime = 30
totalDeliveryTime	The total time needed to deliver food to all retail food stores in Gowanus each day	TBD
trucks	A vehicle making deliveries to the Gowanus district. There is a 1:1 relationship between trucks and drivers	N/A
numberOfTrucks	Total number of trucks that make food deliveries to the Gowanus district each day	51
truckTypes	Truck classification based on the maximum loaded weight of the truck	Classes 2, 3, 7, and 8

We categorized the stores by size, where the store size defines the delivery frequency and delivery time. Here we used an average of 2, 5, 10, and 18 deliveries for the four types of stores: small, medium, large, and extra-large/mega, respectively. The delivery time is estimated at 10 min for small and medium stores and 30 min for large and mega stores.

Considering the working hour limit (8 h in New York State) for each truck driver mandated by the local labor law, the number of deliveries a truck can make also depends on the driver’s delivery time.

The number of trucks are estimated by the ratio of the total delivery time required over driver delivery time. Due to the capacity of different types of trucks, the number of trucks required will depend on the types of trucks used. This is accounted for using the coefficient for each type of truck (Classes 2, 3, 7, and 8) defined in *FTypeOfTrucksnumberOfTrucks* function in Algorithm 1.

#### 3.2.3 Truck Route Calculation

**FIGURE 3:**
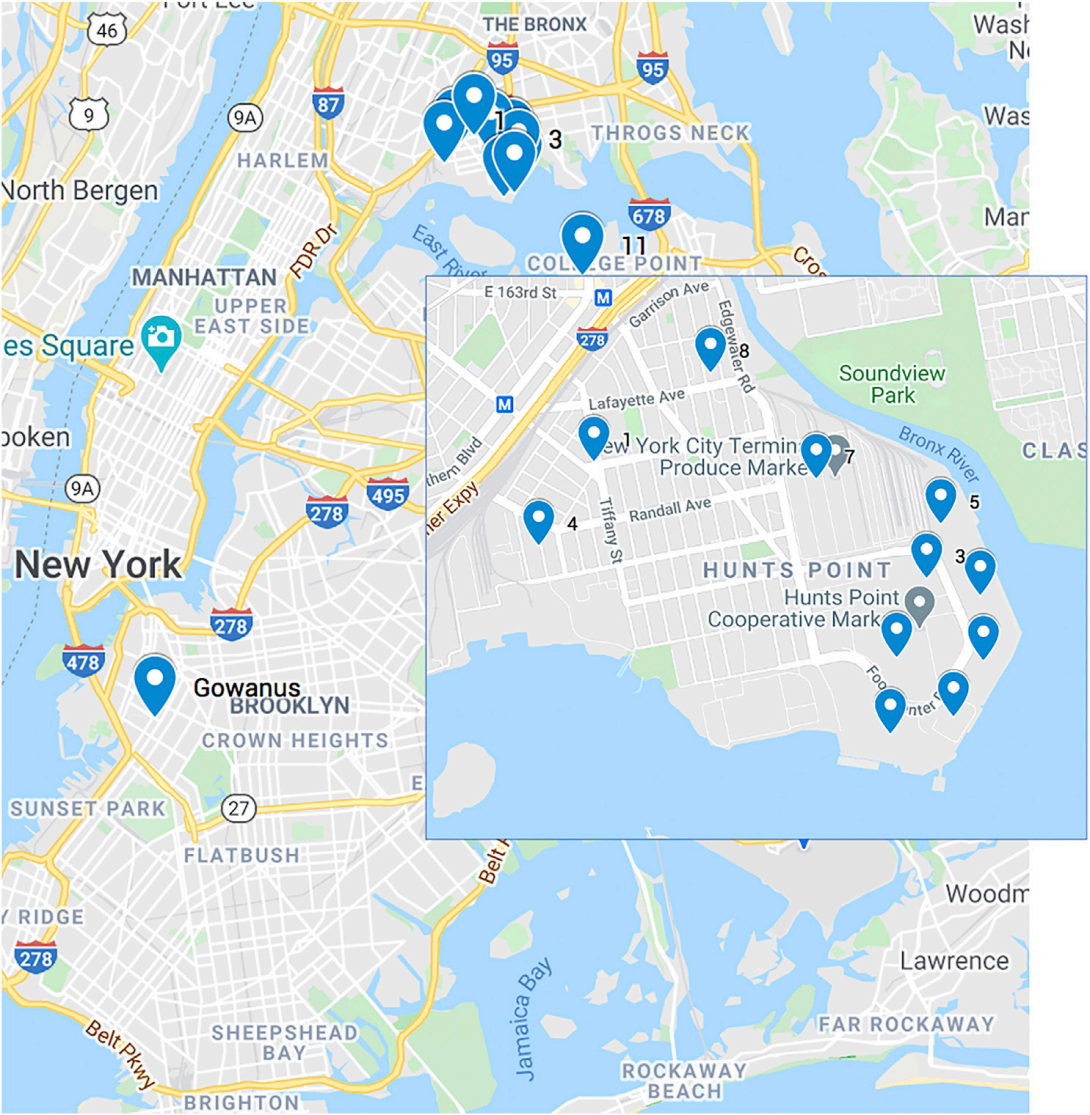
Map of the Hunts Point food distributors and the Gowanus district of NYC.

To study the impact of electric trucks, we compared the energy consumption and GHG emissions of used diesel trucks and electric trucks. Figure 3 shows the Gowanus district and the food distributors on the map. We focus on Hunts Point Meat Market in our case study. We estimate the number of trucks using the ratio of the total delivery time required over driver delivery time. The method is presented in Algorithm 1. This calculation results in four trucks needed to transport food daily from the distribution center to 100 stores in the Gowanus district.

To find the optimal routes between the distribution centers and the multiple stores for each vehicle, we formulate this problem as a Vehicle Routing Problem (VRP) and solve it using Google Operation Research (OR) tools Google (2020). This tool takes the set of inputs shown in Table 3 and provides the optimal route for each vehicle. The source code for the application is available at Elangovan (2021).

**TABLE 3:** Input data for the VRP program.

Data	Description
Distance matrix	An array of distances between locations in meters
Number of vehicles	The number of vehicles in the fleet
Depot	The index of the depot, the location where all vehicles start and end their routes
Demands	Each location has a demand corresponding to the quantity—for example, weight or volume—of the item to be picked up
Vehicle capacity	Each vehicle has a capacity: The maximum quantity that the vehicle can hold. As a vehicle travels along its route, the total quantity of the items it is carrying can never exceed its capacity

The distance between two locations is calculated using the Google Distance Matrix Application Programming Interface (API). This API generates the distance matrix for a set of locations having addresses or latitude and longitude coordinates. The API returns the distance matrix, which consists of rows containing the distance between each pair of locations and it also suggests the route to be followed. We divided the stores into two categories, small and large sizes. We assume that the demand of small-size stores is the same, and that the demand of large sizes is the same. We find the area of these stores, and then set the total capacity of the truck and the area of the stores to estimate the demand in weight for the stores. The weight of the food required by a small store is equal to sum of each area of the small stores divided by the number of small stores. The same calculation is made for finding the food weight demand of large stores. We further evaluated the scenarios where stores have different demands as well as with an increase and decrease of the total demand to show the statistics of the truck scheduled deliveries and traveled distances.

### 3.3 Energy Consumption and GHG Emission

The life cycle is divided into two categories: manufacturing and operations. Usually, the energy consumption and GHG emissions of the manufacturing of a vehicle and electricity are overlooked but they contribute to a fair amount of the total energy consumption and GHG emissions. We consider the manufacturing aspects of an electric truck and diesel truck. The consumed manufacturing energy includes energy consumed for manufacturing a vehicle, Lithium-ion battery production and replacement, supply equipment production and replacement in electric vehicles, and the net energy consumption for end-of-life vehicle recycling of an electric truck. Energy consumption associated with extraction, gathering, transporting and processing of raw materials are included in the manufacturing part of the life cycle analysis. Operational energy consumption is calculated based on the mileage and electricity consumed and on factors such as power plants’ electricity generation efficiency, electric grid transmission efficiency, payload, and efficiency of the electric truck. The unit operational and manufacture consumption as well as GHG emissions formula for the electric trucks are shown in Eqs 1 and 2 respectively. The unit operational energy consumption equation calculates the energy consumption factor. To calculate the energy consumption for a trip, we multiply the energy consumption factor to the weight and distance traveled by the truck. We calculate the GHG emission for the electric trucks. The parameters for energy consumption and GHG emissions for diesel trucks are listed in the Supplementary Material document.

Tables 4, 5 show the definitions of the parameters used in the calculation of the energy consumption and GHG emissions for electric trucks Lee et al. (2013).

**TABLE 4:** Electric truck: Energy consumption parameters.

Parameter	Description	Value
*EC_PPGM_*	Energy consumption of electricity generation and generation mix	0.0039%
η *TM*	Electric grid transmission efficiency	93%
η *Ef*	Efficiency of the electric truck	0.357 km/MJ
*EC_VM_*	Energy consumption for vehicle (electric truck) manufacture	487,000 MJ
*EC_B_*	Energy consumption for Li-Ion battery production	128,000 MJ
*EC_EVSE_*	Energy consumption for electric vehicle supply equipment (EVSE) production	4290 MJ
*EC_Br_*	Energy consumption for Li-Ion battery replacement	128,000 MJ
*EC_EV__SEr_*	Energy consumption for EVSE replacement	4290 MJ
*EC_ELC_*	Net energy consumption of end-of-life vehicle recycling of the electric truck	−122,000 MJ
*LT_EV_*	Lifetime of the truck	240,000 km
*PL_EV_*	Payload of the truck	ton
*EC_ET_*	Total life-cycle energy consumption of the electric truck	MJ/t. km

**TABLE 5:** Electric truck: GHG emissions parameters.

Parameter	Description	Value
*GHG_PPGM_*	GHG emissions from electricity generation and generation mix	0.0039%
η *TM*	Electric grid transmission efficiency	93%
η *Ef*	Efficiency of the electric truck	0.357 km/MJ
*GHG_VM_*	GHG emissions from vehicle (electric truck) manufacture	27,400 *kgCO_2_e*
*GHG_B_*	GHG emissions from Li-Ion battery production	11,300 *kgCO_2_e*
*GHG_EVSE_*	GHG emissions from electric vehicle supply Equipment (EVSE) production	250
*GHG_Br_*	GHG emissions from Li-Ion battery replacement	11,300 *kgCO_2_e*
*GHG_EVSEr_*	GHG emissions from EVSE replacement	250 *kgCO_2_e*
*GHG_ELC_*	Net GHG emission from end-of-life vehicle recycling of the electric truck	−4,660 *kgCO_2_e*
*LT_EV_*	Lifetime of the truck	240,000 km
*PL_EV_*	Payload of the truck	ton
*GHG_ET_*	Total life-cycle GHG emissions from the electric truck	*kgCO_2_e/t. km*

Unit operational energy consumption for electric trucks:

$${OC}_{ET}=\frac{1}{ECPPGM\times \eta TM\times \eta EF\times PL_{EV}}.$$ (1)

Unit manufacture energy consumption for electric trucks:

MCET=ECVMLTEV×PLEV+ECB+ECEVSE+ECBr+ECEVSEr12×LTEV×PLV+ECELCLTEV×PLEV. (2)

Unit operational GHG emissions for electric trucks:

$$OGHG_{ET}=\frac{GHG_{PPGM}}{\eta TM\times \eta Ef\times PL_{EV}}.$$ (3)

Unit manufacture GHG emissions for electric trucks:

MGHGET=GHGVMLTEV×PLEV+GHGB+GHGEVSE+GHGBr+GHGEVSEr12×LTEV×PLEV+GHGELCLTEV×PLEV. (4)

## 4 Results and Discussions

**FIGURE 4:**
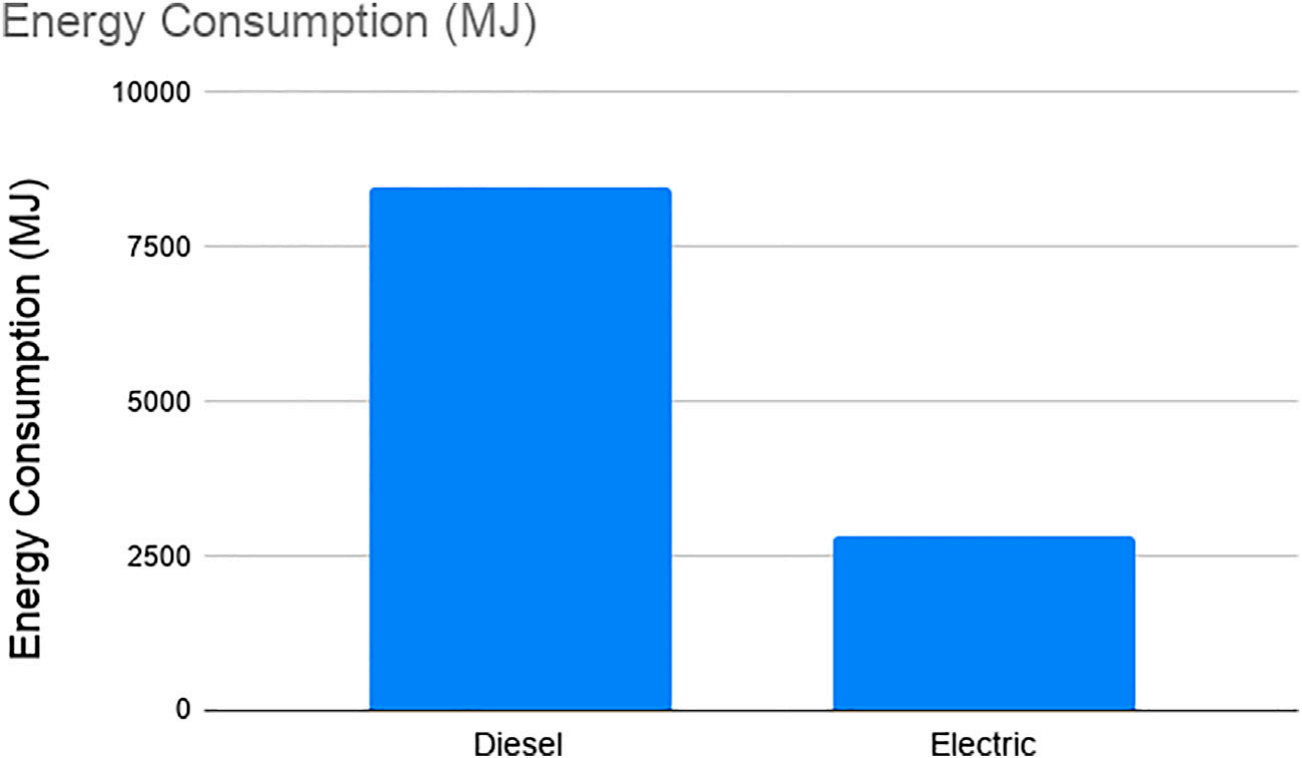
Energy consumption of diesel and electric trucks.

**FIGURE 5:**
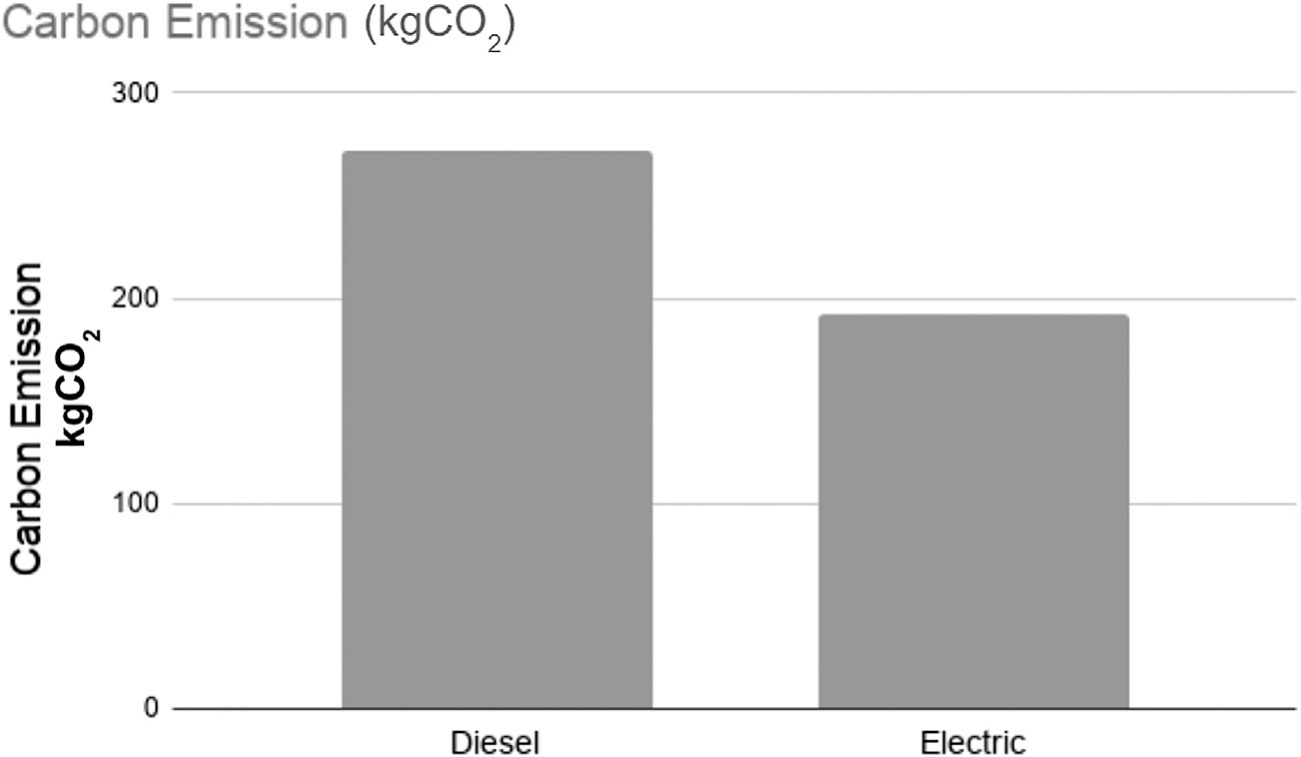
GHG emission of diesel and electric trucks.

In this section, we evaluated the daily energy consumption and GHG emission by diesel and electric trucks that are used to transport food from the distribution center to the food retail stores in Gowanus district. Figures 4, 5 show the daily energy consumption and GHG emission of diesel and electric trucks, respectively. In our case study, for the trips made by the trucks every day to deliver food from the distribution center to the stores, diesel trucks consume 300% more energy and emit 40% more greenhouse gases than electric trucks.

**FIGURE 6:**
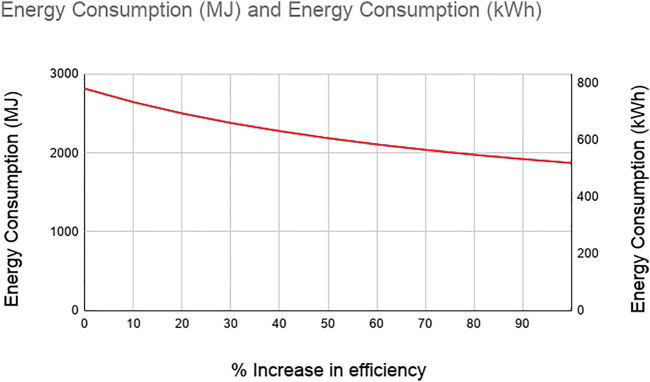
Energy consumption of electric trucks vs. percentage increase in efficiency.

**FIGURE 7:**
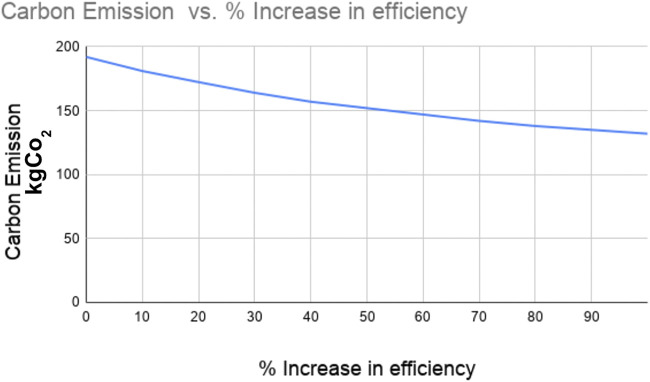
GHG emission of electric trucks vs. percentage increase in efficiency.

Efficiency is the percentage of the amount of electrical energy transferred from the electric grid to power the wheels of the vehicle. It can be measured as the distance traveled per Mega Joules (MJ). The current efficiency of the electric truck is 0.357 km/MJ and when the efficiency increases with the advancement in electric vehicle and battery technology, the energy consumption and GHG emission reduce significantly. Based on the study by Lee et al. (2013), electric vehicles are 117% more efficient than the diesel engine trucks. We evaluated the impact of the efficiency of electric vehicles on their energy consumption and GHG emission by increasing the percentage of efficiency increase. Figures 6 and 7 show the reduction in the energy consumption and GHG emission, respectively with the increase in the efficiency. The figures show that a 90% increase in the efficiency of electric trucks reduces the energy consumption by 34% and the GHG by 33%.

**FIGURE 8:**
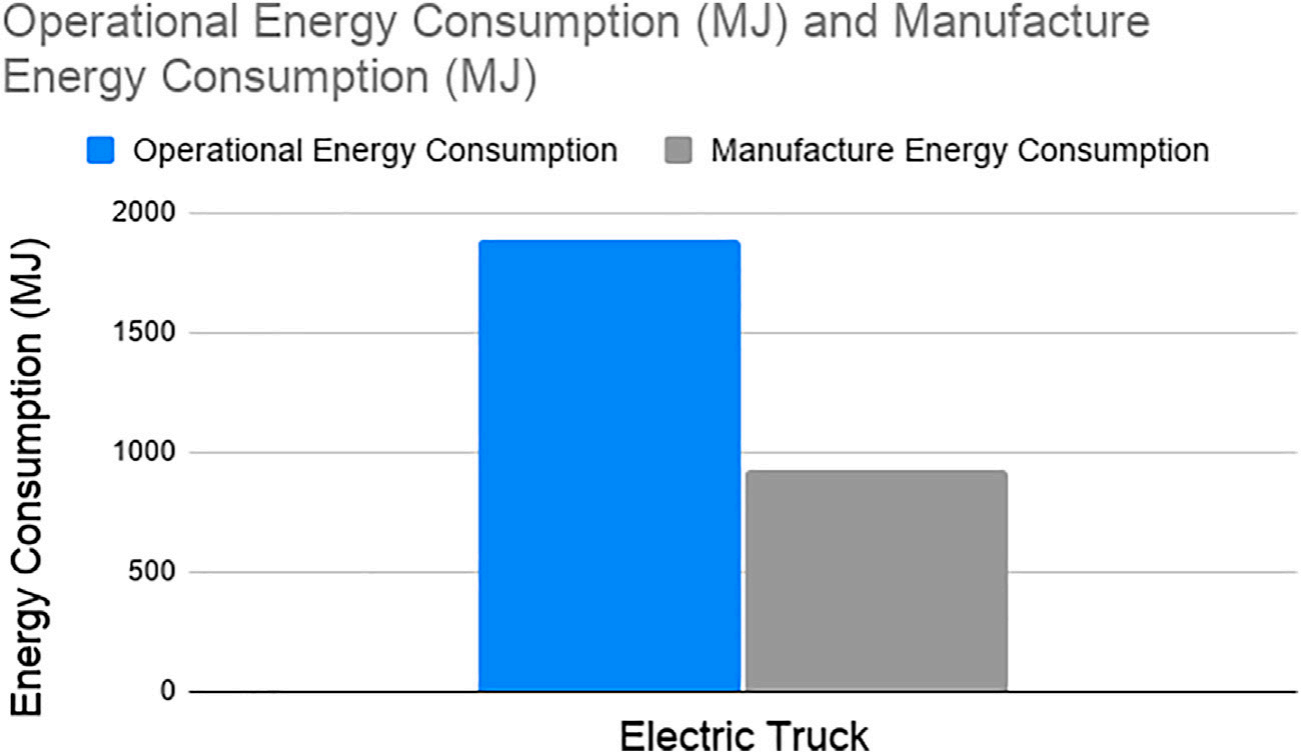
Operational energy consumption vs. manufacture energy consumption for electric trucks.

**FIGURE 9:**
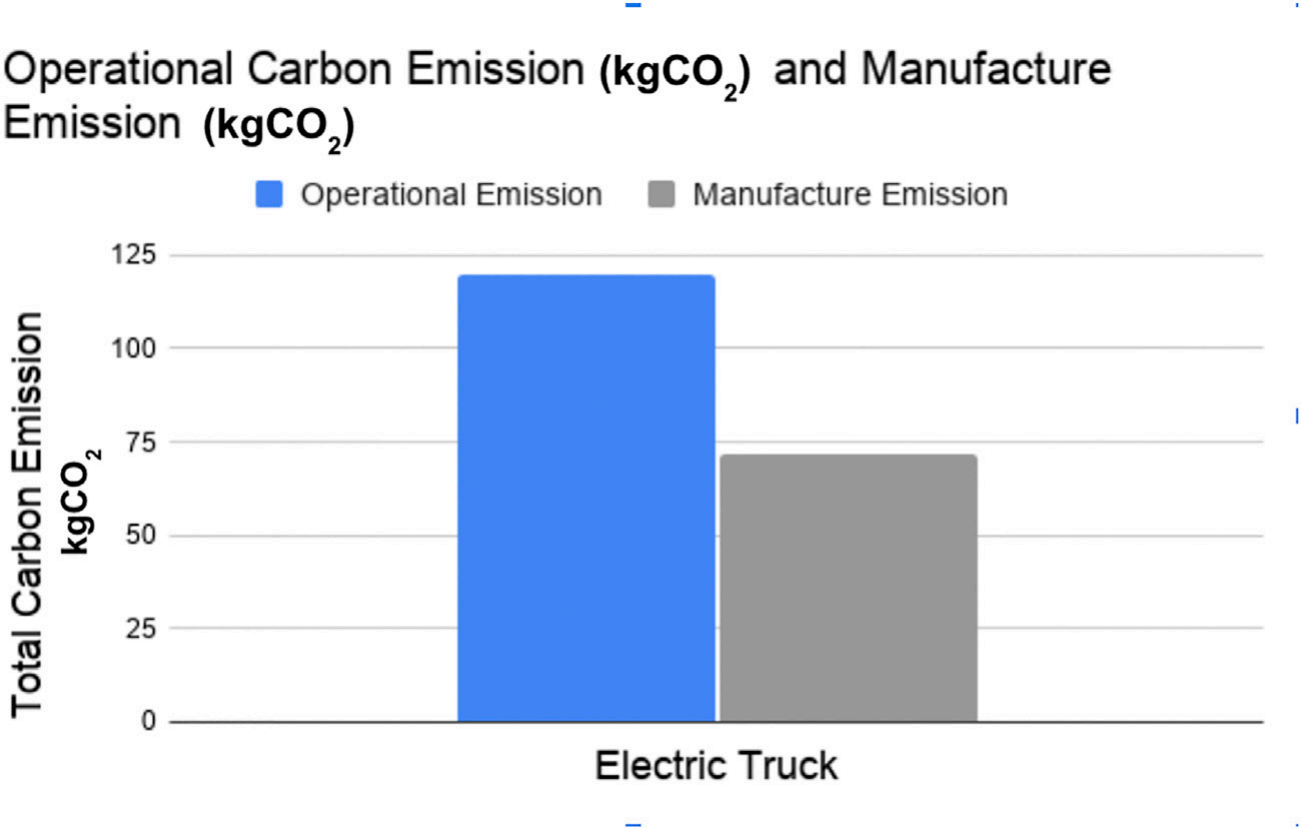
Operational vs. manufacture GHG emission for electric trucks.

Figures 8 and 9 show the operational and manufacture energy consumption and GHG emission of electric trucks, respectively. The energy consumption of the manufacturing phase contributes to 33% of the total energy consumed where manufacturing emissions contribute to 37% of the total GHG emissions by electric trucks. Because the manufacture phase of the trucks contributes a significant amount in energy consumption and GHG emissions, it should be accounted for similar comparative studies.

**FIGURE 10:**
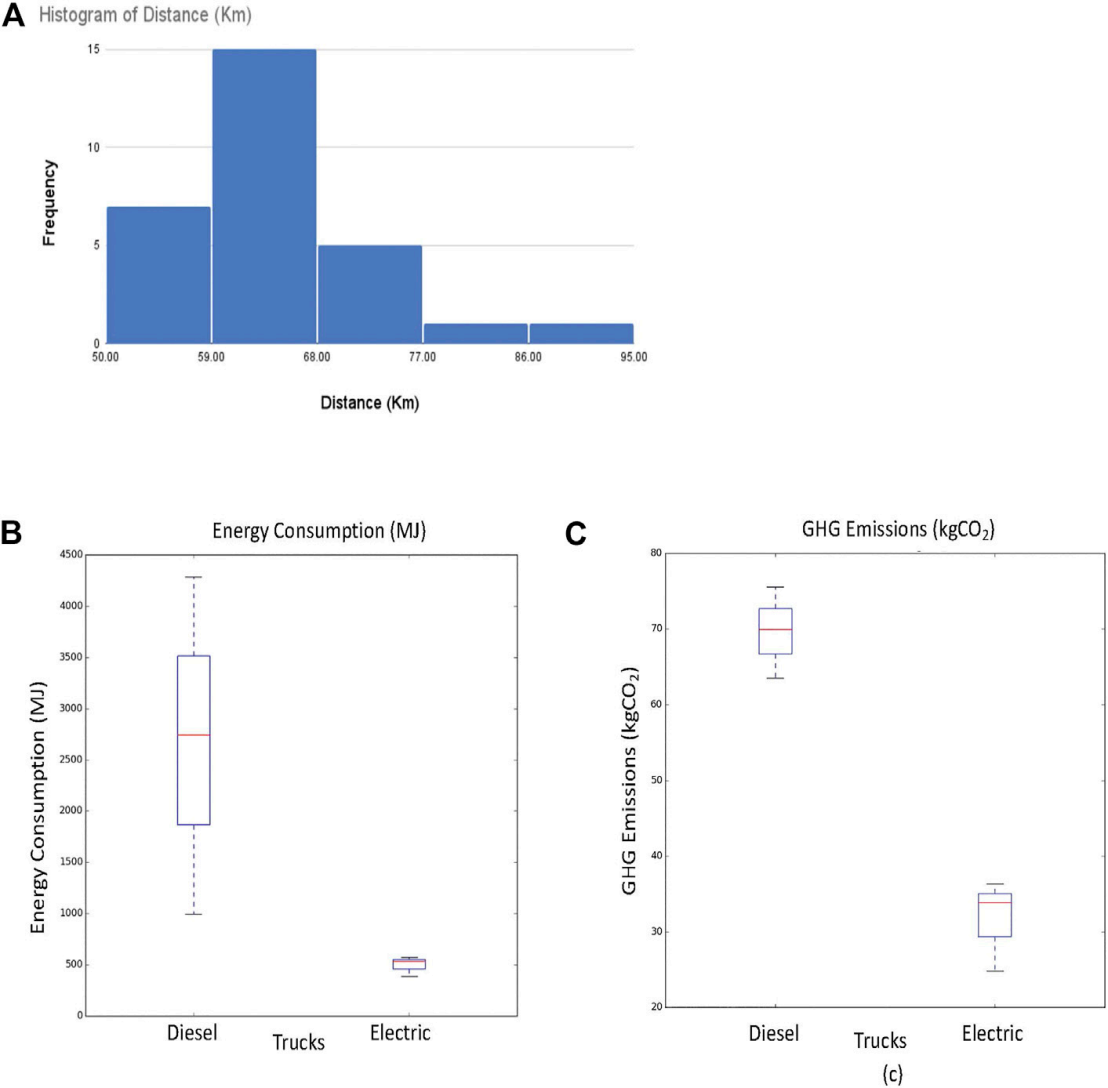
Statistical analysis of electric and diesel trucks with varying store demands. **(A)** Histogram of truck traveled distance. **(B)** Energy consumption of diesel and electric trucks. **(C)** GHG emissions of diesel and electric trucks.

Figure 10 shows a statistical analysis of the evaluated truck deliveries with varying store demands. We considered the cases when the demand is doubled, reduced by half, as well as demand changes in individual stores and showed the histogram of the truck traveled distance (Figure 10A), consumed energy (Figure 10B), and GHG emissions (Figure 10C). The results show that both electric and diesel trucks traveled the same distance and the traveled distance follows a normal distribution. However, diesel trucks consume on average five more times the energy than electric trucks. Electric trucks generate on average half the GHG emission of diesel trucks.

As discussed above, this work uses a representative NYC subset of distributors to estimate the number and types of trucks that serve the Gowanus district. As future work, it is of interest to improve the accuracy of this estimate by including all distributors and their locations. In addition, observational and survey data directly from Gowanus retail food stores and the associated distributors would also enhance the accuracy of the estimated number of trucks.

## 5 Conclusion

We presented a case study on the energy consumption and greenhouse gas emissions of diesel and electric trucks for food distribution in the Gowanus district in Brooklyn, New York. Through an empirical study, we designed a framework that estimates the retail store demands, matches demand to truck deliveries, and schedule truck route to emulate the food delivery from Hunts Point to retail stores at the Gowanus district. The methodology includes the estimation of the number and type of trucks using store information and route selection to address the data gap challenges. We mapped distributors and stores to facilitate the last-mile delivery route planning. To find the optimal routes between these distribution centers and multiple consumer stores for multiple vehicles, we formulated this problem as a Vehicle Routing Problem (VRP) and solved the VRP using Google Operation Research (OR) tools. We studied the life-cycle energy consumption and greenhouse gas emission for the delivery trucks. We compared the daily energy consumption and GHG emissions of diesel and electric trucks for food distribution to local stores. Our results show that diesel trucks consume 300% more energy and emit 40% more greenhouse gases than electric trucks per day.

We also projected the energy consumption and greenhouse gas emissions as functions of increase in efficiency of the electric vehicles with the improvement of vehicle and battery technology. For a 90% increase in the efficiency of electric trucks, the energy consumption is reduced by 34% and the GHG emissions is reduced by 33%. The framework provides an estimate on the impact of electrification of last-mile food delivery. For higher accurate carbon footprint accounting, access to fine-granular data would be necessary.

## Data Availability

The dataset analyzed for this study can be found in the New York City open data repository. https://data.ny.gov/Economic-Development/Retail-Food-Stores/9a8c-vfzj.

## References

[B1] Bates O. Friday A. Allen J. Cherrett T. McLeod F. Bektas T. (2018). Transforming Last-Mile Logistics: Opportunities for More Sustainable Deliveries. In Proceedings of the 2018 CHI Conference on Human Factors in Computing Systems, Montreal, QC, Canada, April 21–26, 2018, 1–14.

[B2] Chang B. (2020). New York City Will Begin Testing a New Fully Electric Garbage Truck — See the Mack LR Electric. Available at: https://www.businessinsider.com/new-york-city-testing-its-first-fully-electric-garbage-truck-2020-9 (Accessed December 10, 2020).

[B3] Division of Food Safety and Inspection (2020). Retail Food Stores. Available at: https://data.ny.gov/Economic-Development/Retail-Food-Stores/9a8c-vfzj (Accessed December 10, 2020).

[B25] Elangovan R. (2021). Route Optimization. Available at: https://github.com/Raghul27/RouteOptimization.

[B4] Elangovan R. Dong Z. (2020). A Case Study of Dietary Pattern Change Impact on Carbon Footprint. Tech. Rep. Food-Energy-Water Nexus. December 5, 2019. New York. American Institute of Chemical Engineers.

[B5] Elsoragaby S. Yahya A. Mahadi M. R. Nawi N. M. Mairghany M. (2019). Analysis of Energy Use and Greenhouse Gas Emissions (GHG) of Transplanting and Broadcast Seeding Wetland rice Cultivation. Energy 189, 116160. 10.1016/j.energy.2019.116160

[B6] Feng W. Figliozzi M. (2013). An Economic and Technological Analysis of the Key Factors Affecting the Competitiveness of Electric Commercial Vehicles: A Case Study from the USA Market. Transportation Res. C: Emerging Tech. 26, 135–145. 10.1016/j.trc.2012.06.007

[B7] Gao Z. Lin Z. Davis S. C. Birky A. K. (2018). Quantitative Evaluation of MD/HD Vehicle Electrification Using Statistical Data. Transportation Res. Rec. 2672 (24), 109–121. 10.1177/0361198118792329

[B8] Ghasemi-Mobtaker H. Mostashari-Rad F. Saber Z. Chau K.-w. Nabavi-Pelesaraei A. (2020). Application of Photovoltaic System to Modify Energy Use, Environmental Damages and Cumulative Exergy Demand of Two Irrigation Systems-A Case Study: Barley Production of Iran. Renew. Energ. 160, 1316–1334. 10.1016/j.renene.2020.07.047

[B9] Goodchild A. V. Ukrainczyk L. (2016). Food Distribution Supply Chain Data Collection: Supply Chain Firm Interviews and Truck Counts. Tech. Rep. Washington (State): Dept. of Transportation. Office of Research and Library.

[B10] Google (2020). Vehicle Routing Problem. Tech. rep., Google. Available at: https://developers.google.com/optimization/routing/vrp (Accessed December 10, 2020).

[B11] Guse C. (2020). MTA Plans to Only Buy Electric Buses Come 2028 as Officials Map Greener Future for NYC Transit. NY. Available at: https://www.masstransitmag.com/bus/vehicles/hybrid-hydrogen-electric-vehicles/news/21220212/ny-mta-plans-to-only-buy-electric-buses-come-2028-as-officials-map-greener-future-for-nyc-transit (Accessed June 10, 2021).

[B12] Khanali M. Akram A. Behzadi J. Mostashari-Rad F. Saber Z. Chau K.-w. (2021). Multi-objective Optimization of Energy Use and Environmental Emissions for walnut Production Using Imperialist Competitive Algorithm. Appl. Energ. 284, 116342. 10.1016/j.apenergy.2020.116342

[B13] Kurien C. Srivastava A. K. (2020). Impact of Electric Vehicles on Indirect Carbon Emissions and the Role of Engine Posttreatment Emission Control Strategies. Integr. Environ. Assess. Manag. 16, 234–244. 10.1002/ieam.4206 31403259

[B14] Lee D.-Y. Thomas V. M. Brown M. A. (2013). Electric Urban Delivery Trucks: Energy Use, Greenhouse Gas Emissions, and Cost-Effectiveness. Environ. Sci. Technol. 47, 8022–8030. 10.1021/es400179w 23786706

[B15] Liimatainen H. van Vliet O. Aplyn D. (2019). The Potential of Electric Trucks - an International Commodity-Level Analysis. Appl. Energ. 236, 804–814. 10.1016/j.apenergy.2018.12.017

[B16] Lv W. Sun Z. Su Z. (2019). Life Cycle Energy Consumption and Greenhouse Gas Emissions of Iron Pelletizing Process in China, a Case Study. J. Clean. Prod. 233, 1314–1321. 10.1016/j.jclepro.2019.06.180

[B17] McCormack E. Ta C. Bassok A. Fishkin E. (2010). Truck Trip Generation by Grocery storesTech. Rep. Seattle, WA: University of Washington.

[B18] Mostashari-Rad F. Ghasemi-Mobtaker H. Taki M. Ghahderijani M. Kaab A. Chau K.-w. (2021). Exergoenvironmental Damages Assessment of Horticultural Crops Using Recipe2016 and Cumulative Exergy Demand Frameworks. J. Clean. Prod. 278, 123788. 10.1016/j.jclepro.2020.123788

[B19] Mostashari-Rad F. Nabavi-Pelesaraei A. Soheilifard F. Hosseini-Fashami F. Chau K.-w. (2019). Energy Optimization and Greenhouse Gas Emissions Mitigation for Agricultural and Horticultural Systems in Northern Iran. Energy 186, 115845. 10.1016/j.energy.2019.07.175

[B20] Nabavi-Pelesaraei A. Kaab A. Hosseini-Fashami F. Mostashari-Rad F. Chau K.-W. (2019). “Life Cycle Assessment (LCA) Approach to Evaluate Different Waste Management Opportunities,” in Book: Advances in Waste-To-Energy Technologies (Taylor & Francis Group), 195–216. 10.1201/9780429423376-12

[B21] NASEM (2021). How We Use Energy- Transportation. Available at: http://needtoknow.nas.edu/energy/energy-use/transportation/.

[B22] NYC (2016). Five Borough Food Supply - New York City Food Distribution and Resiliency Study Results. Tech. Rep. New York City, NY: NYC Economic Development Corporation, NYC Mayor’s office of Recovery and Resiliency. (Accessed December 10, 2020).

[B23] NYC (2019). New york City’s green New deal. Available at: http://onenyc.cityofnewyork.us/ (Accessed December 10, 2020).

[B24] NYCEDC (2016). Five Borough Food Flow. Available at: https://www1.nyc.gov/assets/foodpolicy/downloads/pdf/2016_food_supply_resiliency_study_results.pdf (Accessed December 10, 2020).

[B26] Saber Z. Esmaeili M. Pirdashti H. Motevali A. Nabavi-Pelesaraei A. (2020). Exergoenvironmental-life Cycle Cost Analysis for Conventional, Low External Input and Organic Systems of rice Paddy Production. J. Clean. Prod. 263, 121529 10.1016/j.jclepro.2020.121529

[B27] Salvatore D. Fanti M. P. Mummolo G. Slivestri B. (2017). Externalities Reduction Strategies in Last Mile Logistics: a Review. Tech. Rep. 2017 IEEE International Conference on Service Operations and Logistics, and Informatics (SOLI), Bari, Italy, September 18–20, 2017. 10.1109/SOLI.2017.8121002

[B28] Shi S. Zhang H. Yang W. Zhang Q. Wang X. (2019). A Life-Cycle Assessment of Battery Electric and Internal Combustion Engine Vehicles: A Case in Hebei Province, China. J. Clean. Prod. 228, 606–618. 10.1016/j.jclepro.2019.04.301

[B29] Thornton J. MacArthur J. Barham H. (2018). Electrification of Transport Refrigeration Units for Temperature-Sensitive Freight: U.S. Environmental Protection Agency Region 10 Technical Assistance Case Study. Transportation Res. Rec. 2672, 122–133. 10.1177/0361198118773194

[B30] Yang Z. Wang B. Jiao K. (2020). Life Cycle Assessment of Fuel Cell, Electric and Internal Combustion Engine Vehicles under Different Fuel Scenarios and Driving Mileages in China. Energy 198, 117365. 10.1016/j.energy.2020.117365

[B31] Zhao Y. Onat N. C. Kucukvar M. Tatari O. (2016). Carbon and Energy Footprints of Electric Delivery Trucks: A Hybrid Multi-Regional Input-Output Life Cycle Assessment. Transportation Res. D: Transport Environ. 47, 195–207. 10.1016/j.trd.2016.05.014

[B32] Zhou T. Roorda M. J. MacLean H. L. Luk J. (2017). Life Cycle GHG Emissions and Lifetime Costs of Medium-Duty Diesel and Battery Electric Trucks in Toronto, Canada. Transportation Res. Part D: Transport Environ. 55, 91–98. 10.1016/j.trd.2017.06.019

