# Supplementary material for: Comparative Analysis of Energy Use and Greenhouse Gas Emission of Diesel and Electric Trucks for Food Distribution in Gowanus District of New York City
Source: Front Big Data. 2021 Jul 26;4:693820. doi: 10.3389/fdata.2021.693820 (PMC8350515; doi:10.3389/fdata.2021.693820)
Supplement: Supplementary file 1 [file DataSheet1.PDF]

Supplementary Material

1 APPENDIX

We include all the equations and process in calculating the energy consumption and greenhouse gas emission used to generate results presented in manuscript. The calculation is based on the Georgia Tech report Lee et al. (2013) adapted with New York State data. Table S1 shows the energy consumption parameters for electric trucks.

Operational energy consumption equation for electric trucks:

$$OC_{ET} = \frac{1}{EC_{PPGM} \times \eta_{TM} \times \eta_{Ef} \times PL_{EV}} \tag{S1}$$

Manufacture energy consumption equation for electric trucks:

$$MC_{ET} = \frac{EC_{VM}}{LT_{EV} \times PL_{EV}} + \frac{EC_B + EC_{EVSE} + EC_{Br} + EC_{EVSEr}}{1/2 \times LT_{EV} \times PL_V} + \frac{EC_{ELC}}{LT_{EV} \times PL_{EV}} \tag{S2}$$

Table S2 shows the energy consumption parameters for diesel trucks Lee et al. (2013).

Operational energy consumption equation for diesel trucks:

$$OC_{DT} = \frac{1}{\eta_{UP} \times \eta_{Ef} \times PL_{DT}} \tag{S3}$$

Manufacture energy consumption equation for diesel trucks:

$$MC_{DT} = \frac{EC_{VM} + EC_{VR_{DT}}}{LT_{DT} \times PL_{DT}} \tag{S4}$$

Table S3 shows the GHG emissions parameters for electric trucks Lee et al. (2013).

Operational GHG emissions equation for electric trucks:

$$OGHG_{ET} = \frac{GHG_{PPGM}}{\eta_{TM} \times \eta_{Ef} \times PL_{EV}} \tag{S5}$$

$$MGHG_{ET} = \frac{GHG_{VM}}{LT_{EV} \times PL_{EV}} + \frac{GHG_B + GHG_{EVSE} + GHG_{Br} + GHG_{EVSEr}}{1/2 \times LT_{EV} \times PL_{EV}} + \frac{GHG_{ELC}}{LT_{EV} \times PL_{EV}} \tag{S6}$$

Table S4 shows the GHG emissions parameters for diesel trucks Lee et al. (2013).

Operational GHG emissions equation for diesel trucks:

$$OGHG_{DT} = \frac{GHG_{LC_D}}{\eta_{Ef} \times PL_{DT}} \tag{S7}$$

Manufacture GHG emissions equation for diesel trucks:

$$MGHG_{DT} = \frac{GHG_{VM} + GHG_{VR_{DT}}}{LT_{DT} \times PL_{DT}} \tag{S8}$$

1.1 Tables

Table S1. Electric Truck: Energy Consumption Parameters

Parameter	Description	Value
EC_{PPGM}	Energy Consumption of electricity generation and generation mix	0.0039 %
η_{TM}	Electric grid transmission efficiency	93 %
η_{Ef}	Efficiency of the electric truck	0.357 km/MJ
EC_{VM}	Energy consumption for vehicle (electric truck) manufacture	487,000 MJ
EC_B	Energy consumption for Li-Ion battery production	128,000 MJ
EC_{EVSE}	Energy consumption for Electric Vehicle Supply Equipment (EVSE) production	4290 MJ
EC_{Br}	Energy consumption for Li-Ion battery replacement	128,000 MJ
EC_{EVSEr}	Energy consumption for EVSE replacement	4290 MJ
EC_{ELC}	Net energy consumption of end-of-life vehicle recycling of the electric truck	-122,000 MJ
LT_{EV}	Lifetime of the truck	240,000 km
PL_{EV}	Payload of the truck	ton
EC_{ET}	Total life-cycle energy consumption of the electric truck	MJ/t.km

REFERENCES

Lee, D.-Y., Thomas, V. M., and Brown, M. A. (2013). Electric urban delivery trucks: Energy use, greenhouse gas emissions, and cost-effectiveness. *Environmental science & technology* 47, 8022–8030

Table S2. Diesel Truck: Energy Consumption Parameters

Parameter	Description	Value
η_{UP}	Aggregate upstream efficiency	87.3%
η_{Ef}	Efficiency of the diesel truck	0.093 km/MJ
EC_{VM}	Energy consumption for vehicle (diesel truck) manufacture	540,000 MJ
EC_{VRDT}	Net energy consumption of end-of-life vehicle recycling of the diesel truck	-135,000 MJ
LT_{EV}	Lifetime of the truck	240,000 km
PL_{EV}	Payload of the truck	ton
EC_{DT}	Total life-cycle energy consumption of the diesel truck	MJ/t.km

Table S3. Electric Truck: GHG Emissions Parameters

Parameter	Description	Value
GHG_{PPGM}	GHG Emissions from electricity generation and generation mix	0.0039 %
η_{TM}	Electric grid transmission efficiency	93 %
η_{Ef}	Efficiency of the electric truck	0.357 km/MJ
GHG_{VM}	GHG Emissions from vehicle (electric truck) manufacture	27,400 $kgCO_2e$
GHG_B	GHG Emissions from Li-Ion battery production	11,300 $kgCO_2e$
GHG_{EVSE}	GHG Emissions from Electric Vehicle Supply Equipment(EVSE) production	250
GHG_{Br}	GHG Emissions from Li-Ion battery replacement	11,300 $kgCO_2e$
GHG_{EVSEr}	GHG Emissions from EVSE replacement	250 $kgCO_2e$
GHG_{ELC}	Net GHG Emission from end-of-life vehicle recycling of the electric truck	-4660 $kgCO_2e$
LT_{EV}	Lifetime of the truck	240,000 km
PL_{EV}	Payload of the truck	ton
GHG_{ET}	Total life-cycle GHG emissions from the electric truck	$kgCO_2e/t.km$

Table S4. Diesel Truck: GHG Emissions Parameters

Parameter	Description	Value
GHG_{LC_D}	Life-cycle GHG emissions of diesel fuel	0.09 $kgCO_2e/MJ$
η_{Ef}	Efficiency of the diesel truck	0.093 km/MJ
GHG_{VM}	GHG Emissions from vehicle (diesel truck) manufacture	36,000 $kgCO_2e$
$GHG_{VR_{DT}}$	Net GHG Emissions from end-of-life vehicle recycling of the diesel truck	-6,100 $kgCO_2e$
LT_{EV}	Lifetime of the truck	240,000 km
PL_{EV}	Payload of the truck	ton
GHG_{DT}	Total life-cycle GHG emissions from the diesel truck	$kgCO_2e/t.km$
